# Predictors of career progression and obstacles and opportunities for non-EU hospital doctors to undertake postgraduate training in Ireland

**DOI:** 10.1186/s12960-016-0120-0

**Published:** 2016-06-30

**Authors:** Ella Tyrrell, Conor Keegan, Niamh Humphries, Sara McAleese, Steve Thomas, Charles Normand, Ruairí Brugha

**Affiliations:** Centre for Health Policy and Management, Trinity College Dublin, 3-4 Foster Place, College Green, Dublin 2, Ireland; Department of Epidemiology and Public Health Medicine, Royal College of Surgeons, Dublin, Ireland

**Keywords:** Career progression, International medical graduates, Postgraduate training, Recruitment, WHO Global Code

## Abstract

**Background:**

The World Health Organization’s Global Code on the International Recruitment of Health Personnel urges Member States to observe fair recruitment practices and ensure equality of treatment of migrant and domestically-trained health personnel. However, international medical graduates (IMGs) have experienced difficulties in accessing postgraduate training and in progressing their careers in several destination countries. Ireland is highly dependent on IMGs, but also employs non-European Union (EU) doctors who qualified as doctors in Ireland. However, little is known regarding the career progression of these doctors. In this context, the present study assesses the determinants of career progression of non-EU doctors with particular focus on whether barriers to progression exist for those graduating outside Ireland compared to those who have graduated within.

**Methods:**

The study utilises quantitative data from an online survey of non-EU doctors registered with the Medical Council of Ireland undertaken as part of the Doctor Migration Project (2011–2013). Non-EU doctors registered with the Medical Council of Ireland were asked to complete an online survey about their recruitment, training and career experiences in Ireland. Analysis was conducted on the responses of 231 non-EU hospital doctors whose first post in Ireland was not permanent. Career progression was analysed by means of binary logistic regression analysis.

**Results:**

While some of the IMGs had succeeded in accessing specialist training, many experienced slow or stagnant career progression when compared with Irish-trained non-EU doctors. Key predictors of career progression for non-EU doctors working in Ireland showed that doctors who qualified outside of Ireland were less likely than Irish-trained non-EU doctors to experience career progression. Length of stay as a qualified doctor in Ireland was strongly associated with career progression. Those working in anaesthesia were significantly more likely to experience career progression than those in other specialities.

**Conclusions:**

The present study highlights differences in terms of achieving career progression and training for Irish-trained non-EU doctors, compared to those trained elsewhere. However, the findings herein warrant further attention from a workforce planning and policy development perspective regarding Ireland’s obligations under the Global Code of hiring, promoting and remunerating migrant health personnel on the basis of equality of treatment with the domestically-trained health workforce.

## Background

The lack of education and training opportunities in low- and middle-income countries has been described as a ‘root cause’ of the passive recruitment and migration of health professionals to high-income countries [1]. In an effort to manage this migration pattern and ensure fair and just recruitment and contractual practices, the WHO Global Code of Practice on the International Recruitment of Health Personnel was adopted by the World Health Assembly in May 2010 [2]. Article 4.4 of the Code states that, “*Migrant health personnel should be hired, promoted and remunerated based on objective criteria, such as levels of qualification, years of experience and degrees of professional responsibility on the basis of equality of treatment with the domestically trained health workforce*” [2]. This paper assesses the determinants of career progression of non-European Union (EU) doctors, with particular focus on whether barriers to progression exist for those graduating outside Ireland compared to non-EU nationals who obtain their primary medical qualification in Ireland.

### Career stagnation and gap filling

Research from several countries suggests that international medical graduates (IMGs) experience obstacles to accessing postgraduate training opportunities in their destination countries, contributing to slower and stalled career progression in comparison to their locally-trained colleagues. In the United Kingdom, Young et al. [3] noted that non-EU doctors did not make as many successful applications for specialist registrar 1 posts as their United Kingdom counterparts. IMGs were more likely to be working in non-accredited training posts or to have been appointed to staff and associate specialist group posts, which precluded progression to consultant posts [3]. The United Kingdom Department of Health recognised that there was a perception that, “*doctors in this group, rather than making a positive career choice, have failed elsewhere*” [4]. It is estimated that almost 70 % of doctors taking up these posts are IMGs, with most being unsuccessful in gaining a place on a structured training programme [5].

From 2006, new immigration regulations resulted in the United Kingdom National Health Service only being able to recruit internationally when they could not fill a training post with a United Kingdom or European Economic Area (EEA) graduate, or a refugee doctor [6]. Trewby [7] noted that, “*due to the ruling on work permits, an increase in United Kingdom medical school output and the influx of doctors from the new EEA accession states, it was very difficult for IMGs to receive any training in the United Kingdom; even if their intention following training was to return to practice in their home country*.” Hence, this policy made it more prohibitive for those medical graduates that had trained outside the EEA to join national training programmes.

Other research has shown that IMGs are more likely to fill gaps in specialities that have shortages or those that are hard to fill, such as geriatric medicine in the United Kingdom in the 1980s [8]; psychiatry also relied on IMGs to fill junior and senior posts [9, 10]. Studies in the United States have shown that IMGs tend to practice in areas with doctor shortages, characterised by below average physician-to-population ratios [11]. Further, Mullan et al. [12] noted that IMGs in the United States tend to gravitate towards residency programmes with unfilled positions. Canada has historically relied on internationally-educated health professionals to address shortages in rural and remote locations and hard-to-fill positions [13]. A recent study by Lofters et al. [14], including 483 IMGs, found that many were older physicians who had spent a considerable amount of time and money trying to obtain a medical residency position; IMGs reported the difficulties they experienced in obtaining these positions and securing their future work as doctors in Canada as “*a harsh and unexpected reality*”. Study participants reported that the major obstacle in the process of becoming fully licensed to practice medicine related to difficulties in obtaining postgraduate training. In Australia, the majority of IMGs take up positions as general practitioners in outer metropolitan or rural and remote areas, or work in hospitals as junior medical officers and registrars [15].

McClenahan and Yardumian [16] reported that barriers exist for ethnic minority groups trained within, as well as those trained outside, the United Kingdom who succeed in obtaining senior appointments. They were substantially more likely to work in unpopular parts of the country, less prestigious institutions, or less popular specialities, and were more likely to be in associate specialist, staff grade, or locum posts rather than in consultant posts. The work of McClenahan and Yardumian [16] highlights a distinction, which is addressed herein, between doctors’ nationality and the country of their undergraduate training. The present paper reports and compares the experiences of internationally-trained non-EU doctors who migrated to work in Ireland with those of Irish-trained non-EU doctors.

### Irish context

Ireland’s dependence on IMGs who qualified outside the EU rose from 13.4 % in 2000 to 34 % by 2008, making it the country with the second highest proportion of registered IMGs in the OECD [17]; since then, it has remained at approximately 34–35 % [18, 19]. By 2012, 24 % of non-consultant hospital doctors (NCHDs) 2 in the ‘trainee specialist division’ were IMGs; however, only one fifth of IMGs, many of whom would have arrived in Ireland with several years of postgraduate experience, had progressed on to the ‘specialist division’ of the Register, compared with over 50 % of Irish medical school graduates [19]. Overall, 52 % of IMGs were NCHDs registered in the ‘general division’ of the Register, which contains mainly NCHDs working in what are termed ‘service posts’, which are not part of formal postgraduate training schemes [19]. In total, 74 % of doctors working in these non-training service posts are IMGs [19]. These posts are unpopular with Irish-trained doctors due to the limited career progression they provide. Thus, if most IMGs working in Ireland are not accessing formal postgraduate training and are therefore not progressing to specialist posts (which is the career path for most Irish-trained doctors), the question arises whether this can be attributed to the country of undergraduate training or their nationality/country of origin.

In 2006–2007, Ireland developed and implemented a policy of self-sufficiency in undergraduate and specialist medical training in order to reduce its dependency on IMGs [20, 21], and increased the Irish/EU annual student intake to medical schools from 305 to 725, the estimated medical workforce requirements for self-sufficiency [20, 22], partly through the creation of new graduate entry medical programmes. The target intake was reached by 2011 [23], with graduation in the year 2014–2015. Ireland, however, is unusual, not only in its heavy reliance on the recruitment of IMGs, but also in its high intake of students from outside the EU: 400 non-EU medical graduates are expected to graduate from Irish medical schools in 2014–2015 [personal communication, National Doctors Training and Planning, Ireland], comprising 35 % of the overall graduate numbers. In other high-income countries, non-EU nationals reportedly account for only 10 % of the total medical school student body [20]. In Irish medical schools, enrolling non-EU nationals is seen as an income-generating activity, as these students pay higher fees which in turn helps to compensate for a combination of low government subsidies and fees charged to EU nationals [24].

It is generally assumed that these non-EU nationals return to their countries of origin after graduation from Irish medical schools [20]. However, despite 92 % of the 684 intern posts in 2014 being filled by Irish/EU graduates [personal communication, ibid], some of the non-EU national graduates remain in Ireland for their internship (the pre-registration year after qualifying from medical school). Additionally, in recent years, the rate of emigration of Irish-trained Irish doctors, which has historically been high (though not accurately quantified), is believed to have increased. In mid-2011, just under half of the doctors who completed an internship (the year of practice prior to full registration) left the country [25]. This scale of doctor emigration is widely seen as exerting further pressure on employers to recruit IMGs, actively as well as passively, to fill these gaps.

Research across several high-income countries shows that IMGs experience challenges with career progression. This may involve being appointed to grades which have no systematic training or career progression, making it difficult to access higher training schemes and achieve permanent posts. In response, many IMGs choose to work in specialties that are under-subscribed and accept (or are obliged in some countries) to work in less attractive locations. Ireland still has 900 service posts that Irish doctors will not apply for because they offer no formal training or career progression, and is highly dependent on IMGs taking up these posts. Ireland has a unique cohort of non-EU doctors: some migrate to Ireland for undergraduate training, while most come as qualified doctors, seeking postgraduate training [26, 27]. The present analysis compares the career progression experiences of these two groups of non-EU doctors working in Ireland based on responses to an online survey and with particular focus on whether barriers to progression exist for those graduating outside Ireland. Previous research based on in-depth interviews with 37 non-EU doctors working in Ireland [26, 27] indicated that most believed that the demand for non-EU migrant doctors in Ireland was driven by the need to fill specific posts. Respondents felt that their hopes for career progression and postgraduate training in Ireland were unrealised and that they were becoming de-skilled; as a result, most respondents were actively considering onward migration [26, 27].

The present paper aims to compare the differences in career progression of non-EU doctors who obtained their undergraduate medical degree in Ireland to non-EU doctors who trained outside Ireland, as well as to ascertain the key factors that predict substantial career progression for non-EU doctors in Ireland and to assess Ireland’s policy responses to Article 4 of the Code.

## Methods

### Data

This paper analyses quantitative data from the Doctor Migration Project (2011–2013) [26, 27], which comprised a qualitative phase (in-depth interviews with 37 non-EU doctors) followed by a quantitative online survey of non-EU doctors registered with the Medical Council of Ireland (MCI). The survey was constructed using the online tool Survey Monkey. The sampling frame of interest included non-EU graduates of Irish medical schools, non-EU citizens who graduated from EU medical schools, and non-EU citizens who graduated outside the EU (which represented most non-EU doctors in Ireland). All 4,965 non-EU doctors registered with the MCI with a valid email address (96.5 % of those eligible to participate in the study) comprised the sampling frame.

A sample size of 357 was sought to provide a ±5 % margin of error based on the overall non-EU doctor population. In line with previous surveys on migrants in the Irish context, we anticipated a low response rate [28, 29] of approximately 20 %. The MCI emailed 3,009 non-EU doctors on behalf of the research team, inviting them to complete the online survey and received 483 responses, of which 366 were fully completed – the data used in this paper only relate to these fully completed responses.

In terms of the inclusion and exclusion criteria, the analysis of the data collected for this paper was conducted on the responses of 231 non-EU doctors whose first post in Ireland was not permanent (i.e. non-consultants), as the focus of this study was the career progression of hospital doctors. In the overall sample, four doctors had been actively recruited, while the remainder were passively recruited. However, doctors actively recruited to Ireland [30] did not comply with the inclusion criteria for this analysis as they did not experience career progression due to rules prohibiting them from moving to another post.

Medical training in Ireland involves four principal stages: medical student, intern, initial specialist training, and higher specialist training. For the purposes of this analysis, a first medical job included all grades from intern to specialist registrar.

### Exclusion criteria

Respondents who were appointed directly to consultant or general practitioner (GP) posts as their first post upon medical employment in Ireland were excluded, as the model for career progression was based on promotion of at least two steps or attainment of a consultant post.

### Inclusion criteria

Current medical post, at the time of the survey, included all grades from intern to consultant. The following grades were excluded from the analysis: GP trainee, GP, those not working or retired, and those not living in Ireland.

The total number of respondents included in this analysis was 231. Table 1 shows the variables included in the model and a description of how they were coded.

**Table 1:** Variables included in the model

Variable	Definition
Age	Age in years
Sex	Male/Female
Country of qualification	Broken down by the most commonly cited countries (Ireland, India, Nigeria, Pakistan, and Sudan) with the remainder categorised as ‘Other’
Year of qualification	Number of years since primary degree was obtained
Clinical speciality	Based on first medical job and categorised into the five most commonly cited specialities: Medicine, Anaesthesia, Surgery, Paediatrics, Psychiatry, with the remainder categorised as ‘Other’
Irish citizenship status	This variable is coded as 'Yes', 'No' or 'Already hold citizenship'
Applications for recognised training scheme	The responses are coded as ‘Yes’ or ‘No’ Note: ‘Yes’ is aggregated based on: Yes-unsuccessful, Yes-currently on scheme and Yes-completed
Length of stay	Length of time in Ireland^a^

^a^Based on first year of arrival for respondents qualified outside Ireland, while for Irish-trained, it is based on their year of qualification

Respondents were asked to identify the grade of their first medical job in Ireland as well as their current medical job. For this analysis, we defined career progression as a binary variable – either limited progression (0) or substantial progression (1). Limited progression was defined as no progression or increasing one grade, whereas substantial progression was defined as increasing two or more grades from first medical job in Ireland to current medical job in Ireland, or obtaining a consultant post. The determinants of career progression were then modelled by way of logistic regression analysis.^3^ That is,

$$ Prob\ \left( Substantial\kern0.5em  Progression=1\Big|X\right)=G\left(X\beta \right) $$

Where G(.) is the logistic function and $\beta$ is a set of parameters associated with the vector of explanatory variables, $X$ (Table 1). In this analysis we focused on odds ratios (OR) as the measure of association (see Table 7). Odds ratios represent the exponentiated value of the estimated regression coefficients. ORs capture the ratio of odds for a one-unit change in an explanatory variable.

The Ethics Committee of the Health Policy and Management and Centre for Global Health Research at Trinity College Dublin reviewed and approved the study.

## Results

Table 2 illustrates the main descriptive statistics. The mean age of all respondents was 40 years, while 68 % were male. The most frequently cited country of qualification was Pakistan (22 %), followed by Ireland (22 %) and other (19 %). The sample consisted only of doctors who qualified in non-EU countries (n = 181) and non-EU doctors who qualified from medical schools in Ireland (n = 50), which provides a useful comparative category to assess career progression, distinguishing country of origin from country of medical training.

**Table 2:** Descriptive statistics

		*n* (%)	Mean (Median)	SD (Min/Max)
Age, years			39.9 (38.0)	8.7 (24/68)
Sex	Male	154 (67.5		
	Female	74 (32.5)		
Country primary medical degree was obtained	Ireland	50 (21.6)		
	India	32 (13.9)		
	Nigeria	32 (13.9)		
	Pakistan	51 (22.1)		
	Sudan	22 (9.5)		
	Other	44 (19.0)		
Years since qualification			15.7 (14.0)	9.1 (2/54)
Clinical speciality	Medicine	69 (30.4)		
	Anaesthesia	28 (12.3)		
	Surgery	39 (17.2)		
	Paediatrics	16 (7.0)		
	Psychiatry	36 (15.9)		
	Other	39 (17.2)		
Application for Irish citizenship	Yes	58 (25.1)		
	No	99 (42.9)		
	Already hold citizenship	74 (32.0)		
Application for a place on a recognised training scheme	Yes	188 (82.1)		
	No	41 (17.9)		
Length of stay in Ireland as a qualified doctor, years			10.1 (9.0)	6.9 (1/54)

The mean number of years since qualification was 15.7 years. The most frequently cited speciality was medicine (30 %), followed by other (17 %) and surgery (17 %); 25 % of respondents had applied for Irish citizenship at the time of survey, while 32 % currently held citizenship and 43 % of respondents had not applied. Most respondents (82 %) had applied for a place on an official training scheme and had either completed, applied unsuccessfully, or were currently on a scheme. The mean length of time that respondents had worked in Ireland as a qualified doctor at the time of survey was 10.1 years. Overall, 44.2 % of doctors had progressed more than one level (Table 3).

**Table 3:** Career progression frequencies

	*n* (%)
Limited career progression (no career progression or one level increase)	129 (55.8)
Substantial progression	102 (44.2)
Total	231 (100)

Table 4 shows the change in medical grade from first medical job to current medical grade in Ireland. Internship (the first post after graduation) was reported by 24 % of respondents, 92 % of whom had obtained their primary medical qualification in Ireland. Senior house officer grade, the next step after internship, was reported by 59 % as their first medical post, while only 16 % remained at this grade in their current medical grade. Further, 14 % of respondents cited their first medical post as a registrar and 40 % stated that this was their current grade, indicating that career progression occurred into this category. Only 1 % of respondents first began working in Ireland as a senior registrar, while 11 % cited this as their current grade. Only 2 % of respondents reported that their first medical post was as a specialist registrar, with 11 % in this advanced training grade at the time of the survey. Of the 18 % of respondents who had progressed to a consultant post in Ireland, most had qualified as doctors in Pakistan (42 % 18/42), followed by India (24 % 10/42).

**Table 4:** First medical grade in Ireland to current grade

Country of qualification		Ireland	India	Nigeria	Pakistan	Sudan	Other	Total
		*n* (%)	*n* (%)	*n* (%)	*n* (%)	*n* (%)	*n* (%)	*n* (%)
Intern	First grade	46 (92)	0 (0)	2 (6)	1 (2)	1 (5)	6 (14)	56 (24)
	Current grade	3 (6)	0 (0)	0 (0)	0 (0)	0 (0)	4 (9)	7 (3)
Senior House Officer	First grade	2 (4)	18 (56)	29 (91)	41 (80)	17 (77)	29 (66)	136 (59)
	Current grade	10 (20)	3 (9)	4 (13)	7 (14)	2 (9)	12 (27)	38 (16)
Registrar	First grade	0 (0)	11 (34)	1 (3)	8 (16)	4 (18)	8 (18)	32 (14)
	Current grade	15 (30)	12 (38)	19 (59)	18 (35)	14 (64)	15 (34)	93 (40)
Senior Registrar	First grade	0 (0)	2 (6)	0 (0)	0 (0)	0 (0)	0 (0)	2 (1)
	Current grade	3 (6)	3 (9)	5 (16)	6 (12)	4 (18)	4 (9)	25 (11)
Specialist Registrar	First grade	2 (4)	1 (3)	0 (0)	1 (2)	0 (0)	1 (2)	5 (2)
	Current grade	14 (28)	4 (13)	0 (0)	2 (4)	1 (5)	5 (11)	26 (11)
Consultant	First grade	0 (0)	0 (0)	0 (0)	0 (0)	0 (0)	0 (0)	0 (0)
	Current grade	5 (10)	10 (31)	4 (13)	18 (35)	1 (5)	4 (9)	42 (18)

Table 5 shows the length of stay in Ireland from the first year of arrival,^4^ with the mean varying from 8.7 to 11.9 years, indicating that the non-EU doctors in this sample had remained in Ireland for a significant length of time. Respondents who were in Ireland for the longest period had qualified in India (mean 11.9 years), followed by Pakistan (mean 11.8 years), Nigeria (mean 10.1 years), other (mean 8.7 years), and Sudan (mean 8.6 years). Those who obtained their basic medical degree in Ireland had been working as qualified doctors, on average, for 9.2 years. The mean age at arrival was between 30 and 33 years, which was similar across all countries except for Ireland, which was 23 years since Irish-trained doctors would have entered the medical workforce directly after graduation.

**Table 5:** Length of stay and age at arrival

	Country of primary qualification					
	Ireland	India	Nigeria	Pakistan	Sudan	Other
	Mean (SD)	Mean (SD)	Mean (SD)	Mean (SD)	Mean (SD)	Mean (SD)
Length of stay in Ireland as a qualified doctor, years	9.2 (7.8)	11.9 (6.3)	10.1 (3.7)	11.8 (8.1)	8.6 (3.8)	8.7 (7.5)
Current age, years	32.5 (4.5)	44.5 (8.9)	40.9 (5.2)	42.8 (8.9)	40.8 (7.5)	40.2 (9.8)
Age at arrival, years	23.3 (6.9)	32.6 (4.6)	30.8 (3.4)	30.9 (5.4)	32.2 (5.3)	31.5 (5.9)

Before modelling was conducted, all variables were examined to test their (unadjusted) associations with career progression, assessing their statistical significance before controlling for other variables (Table 6). Percentages show the breakdown of variables categorised as experiencing either limited or substantial career progression. Doctors who progressed in their careers were over 4 years older, on average, than those who had limited  career progression in Ireland (38 years, limited progression vs. 42.18years, substantial progression). Those who progressed were more likely to have qualified in Ireland (12 %, limited progression vs. 33 %, substantial progression) and were longer qualified (13.6 vs. 18 years). Doctors who had substantial progression were more likely to hold Irish citizenship (25 %, limited progression vs. 41 %, substantial progression) or to have applied for citizenship (21 %, limited progression vs. 30 %, substantial progression). Moreover, they were more likely to have applied for a place on an official training scheme in Ireland (77 %, limited progression vs. 89 %, substantial progression). Those who experienced substantial career progression had spent a longer period in Ireland compared to those who experienced limited career progression (13.6 vs. 7.4 years).

**Table 6:** Descriptive statistics by career progression

Variable	0 (Limited career progression)		1 (Substantial career progression)		Statistical significance (*P* value)
	n (%)	Mean (SD)	n (%)	Mean (SD)	
Age		38.01 (7.79)		42.18 (9.26)	<0.01
Sex					
Male	86 (67.7)		68 (67.3)		
Female	41 (32.3)		33 (33.3)		
Years since qualification		13.55 (7.94)		18.42 (9.67)	<0.01
Length of stay in Ireland as a qualified doctor		7.40 (4.98)		13.59 (7.53)	<0.01
Country of qualification					
Ireland	16 (12.4)		34 (33.4)		<0.01
India	17 (13.2)		15 (14.7)		
Nigeria	23 (17.8)		9 (8.8)		<0.05
Pakistan	24 (18.6)		27 (26.5)		
Sudan	16 (12.4)		6 (5.9)		
Other	33 (25.6)		11 (10.8)		<0.01
Medical speciality					
Medicine	45 (35.7)		24 (23.8)		
Anaesthesia	10 (7.9)		18 (17.8)		<0.05
Surgery	26 (20.6)		13 (12.9)		
Paediatrics	9 (7.1)		7 (6.9)		
Psychiatry	20 (15.7)		16 (15.8)		
Other	16 (12.7)		23 (22.8)		<0.05
Citizenship status					
Applied	27 (20.9)		31 (30.4)		
No	70 (53.8)		29 (28.4)		<0.01
Already hold	32 (25.4)		42 (41.2)		<0.01
Training scheme status					
Yes	97 (76.4)		91 (89.2)		<0.05
No	30 (23.6)		11 (10.8)		<0.05

### Logistic regression model

Table 7 shows the results of the logistic regression analysis. Country of qualification was significantly associated with career progression, independently of other factors including length of time in Ireland. Doctors who qualified in India (OR = 0.07; *P* <0.05), Nigeria (OR = 0.07; *P* <0.05), Pakistan (OR = 0.20; *P* <0.05), and Sudan (OR = 0.07; *P* <0.05) were all considerably less likely to progress compared to non-EU doctors trained in Ireland. As one would expect, length of time in Ireland as a qualified doctor was a statistically significant predictor of career progression and the magnitude of this effect was significant. For every additional year that a respondent had been in Ireland, the likelihood of career progression increased by approximately 54 % (OR = 1.54; *P* <0.01). Understandably, those who had applied for an official training scheme were considerably more likely to experience career progression than those who had not. Choice of medical speciality was not associated with career progression, with one exception; non-EU doctors who specialised in anaesthesia were nearly 15 times more likely to experience career progression than those in other specialties (OR = 14.69; *P* <0.01). In contrast, there was no evidence that age, sex, citizenship status, or year of qualification had any impact on career progression.

**Table 7:** Logistic regression model

Variable	Odds ratio (95 % confidence interval)	*P* value
Age	0.85 (0.70–1.05)	0.127
Sex (*Ref = Male*)	0.71 (0.29–1.77)	0.468
Year of qualification	1.07 (0.868–1.31)	0.535
Length of stay in Ireland as a qualified doctor	1.54 (1.27–1.86)	<0.001
Country of qualification (*Ref = Ireland*)		
India	0.07 (0.01–0.38)	0.002
Nigeria	0.07 (0.01–0.36)	0.002
Pakistan	0.20 (0.04–0.93)	0.04
Sudan	0.07 (0.03–0.68)	0.004
Other	0.13 (0.03–0.68)	0.015
Medical speciality (*Ref = Medicine*)		
Anaesthesia	14.69 (3.33–64.84)	0.001
Surgery	1.18 (0.31–4.48)	0.813
Paediatrics	2.87 (0.68–12.07)	0.15
Psychiatry	1.38 (0.36–5.32)	0.641
Other	1.96 (0.61–6.23)	0.256
Citizenship status (*Ref = Citizen*)		
No	0.50 (0.18–1.35)	0.131
Training scheme status (*Ref = Applied*)		
Have not applied for a training scheme	0.11 (0.03–0.45)	0.002
Constant	17.19	0.307

## Discussion

### Career progression prospects for non-EU doctors in Ireland

This paper has revealed the key predictors of career progression for non-EU doctors working in Ireland. Results show that most doctors did experience some progression whilst working in Ireland. However, country of qualification had an impact on the likelihood of promotion. Particularly, relative to non-EU doctors trained in Ireland, those who qualified outside of Ireland were less likely to experience career progression. This could be related to the significant variation in the structure and quality of undergraduate medical education across countries. As noted by Karle [31], *"regional and cultural differences in medical education traditions and theories, disease preponderance, clinical guidelines, available resources, doctor-patient relationships etc, lead to a considerable variation in the nature of medical education offered in the various institutions worldwide."*     

Length of stay as a qualified doctor in Ireland was strongly associated with career progression. Irish-trained non-EU doctors were at an earlier stage of their career (8–10 years younger) than non-EU trained doctors; findings show that Irish-trained doctors had further to progress in their medical career and had progressed through more stages in postgraduate training. In Ireland, achieving a post on a postgraduate training programme is competitive; therefore, slow progression through the required stages is likely to be viewed negatively in the assessment and rapid progression is usually seen as a marker of high performance, increasing the candidate’s competitiveness at interview. ‘Length of time as a qualified doctor’ and ‘country of qualification’ were both independently statistically significantly associated with career progression in logistic regression. This could be related to the formal and informal networks and links that doctors accumulate while completing their undergraduate medical education in Ireland, making it easier for them to obtain an interview/job compared to doctors who have no experience of the Irish health system.

As reported elsewhere [3, 8, 10], non-EU doctors working in Ireland have a better chance of promotion in specialities facing greater shortages [32]. Within our sample, those working in anaesthesia were significantly more likely to experience career progression than those in other specialities. This shortage of anaesthetists was highlighted in a recent media article [33], which noted that the College of Anaesthetists of Ireland believe that, “*a long-term failure to ensure Ireland has enough anaesthetists in its hospitals is increasing the risk of serious medical mistakes during surgery and delaying vital treatment for patients*.”

As anticipated, applying for a training scheme improves career prospects for non-EU doctors. However, it is unclear whether doctors who do not apply for a training scheme occupy posts where training is not available (‘service posts’) or whether they simply do not apply for training schemes. A possible contributing factor for non-Irish trained doctors experiencing difficulties in career progression is a policy prohibiting doctors from some non-EU countries accessing postgraduate training in the Trainee Specialist Division.^5^ In late January 2015, it was reported that doctors from certain non-EU countries were prohibited from accessing training posts under a legislative change [34], which stated that only non-EU doctors from Australia, New Zealand, Malaysia, Sudan, South Africa (with an internship after 2006), and Pakistan (with an internship after 2008) can enter training programmes or obtain specialist posts. According to the report, non-EU doctors from other countries cannot apply for training posts that lead to specialisation and consultant positions, regardless of their qualifications or experience.

Citizenship status was significantly associated with career progression in the initial analysis (Table 6), but not in the logistic regression analysis. Immigrants can only apply for citizenship if they have lived in Ireland for 5 years; therefore, this variable would have been highly correlated with the length of time in Ireland, which was significantly associated with career progression. Those who are more settled may be more likely to apply for citizenship, which could assist with better career progression. On the other hand, citizenship might be a result of career progression, i.e. those who have progressed to a certain level apply for citizenship in order to secure their future status in Ireland. An earlier qualitative phase of this study reported the opportunity to obtain citizenship as a factor influencing non-EU doctors’ decision to remain in Ireland [27].

### Policy responses - the code in action

Ireland illustrates the type of country where the Code has particular relevance, in that it has a highly permeable health system and is an important source and destination country for migrating doctors. The following section assesses Ireland’s policy responses to Article 4 of the Code. The Code, which promotes the principle of equal treatment of migrant and domestic health personnel, is particularly important in relation to hiring, promotion and remuneration practices (Article 4.4), and provides opportunities and incentives to strengthen professional education, qualifications and career progression (Article 4.6).

Given the history of international recruitment of doctors to Ireland since 2000, and particularly over the last 5 years, it might not be obvious why Ireland is cited as an exemplar of good practice for its achievements in addressing the challenges of health worker migration and upholding of the principles of the Code [35]. However, policy level and national health workforce responses since 2012 help explain this international attention and, while efforts to build training self-sufficiency commenced in 2006, some of the credit for recent responses can be attributed to the Code, which resulted in national stakeholder organisations utilising it as a way of driving efforts to address the challenges associated with Ireland’s heavy reliance on IMGs.

Article 4.4 of the Code ends with the statement, “*Recruiters and employers should provide migrant health personnel with relevant and accurate information about all health personnel positions that they are offered*” [2]. For doctors, postgraduate training opportunities are crucial for career advancement and are a basic requirement for specialisation. Therefore, it is important that doctors who are planning to migrate are aware of the training and career obstacles, as well as the opportunities, likely to be faced in the destination country of their choice, thus ensuring that their expectations are aligned with what the destination country has to offer. This has been highlighted in previous research [36], where, from the overall sample of 366 doctors, 55 respondents (24 %; 51 of whom had migrated passively) reported that they had received inaccurate information about the types of opportunities available to them in Ireland.

As previously noted, Ireland now prohibits access to postgraduate training for doctors from certain countries. Whether or not this policy is compliant with the principles of the Code, which states that migrant health workers should enjoy equal opportunities to domestically-trained graduates, is debatable. In particular, Article 4.4 includes a ‘get-out’ clause by stating that, “*equality of treatment with the domestically trained health workforce*”, along with the other injunctions of the clause, are “*… to the extent possible under applicable laws*”. Although the Code does not require destination countries to provide postgraduate training to IMGs, the experiences of IMGs in Ireland do reinforce the importance of health professionals having a clear understanding of the opportunities and obstacles to training before they migrate.

A Strategic Review of Medical Training and Career Structures in Ireland, was published by the Department of Health in 2014. The report sets out a number of high-level recommendations relating to training and career pathways for doctors with a view to improving graduate retention in the public health system, planning for future service needs, and realising maximum benefit from investment in medical education and training [37, 38]. Among the recommendations to address a range of barriers and issues relating to the recruitment and retention of doctors in the public health system, one was specifically focused on resolving the issue of reliance on service posts to staff the health service, with a disproportionate number of non-EU doctors, who have not obtained a place on a higher specialist training scheme or achieved consultancy, occupying such posts. The structure of the Irish health system means that there are more doctors in training than there are consultant posts for them to work towards [23]. The Workforce Planning Unit of Ireland’s Department of Health now coordinates joint efforts, including a committee for monitoring implementation of the recommendations of the Strategic Review, which includes representatives of the main stakeholders.

Ireland’s new policies towards health workforce development reflect how the Code is acting as an agent of change in Ireland through helping to identify win-win strategies that benefit both source and destination countries. More generally, the Code has helped to focus decision-makers by shedding light, internationally, on what have been long-standing weaknesses in Ireland’s medical workforce practices. National stakeholders have been able to use Ireland’s obligations under the Code as a way to drive changes that are in the interests of the Irish health system and Irish doctors, as well as those countries, such as Pakistan, who wish to avail of postgraduate training for their doctors. However, more work is required in this area in order to ensure Ireland is meeting its commitment to the Code and its obligations under Article 4. Consideration should be given to the likely career progression that Ireland can offer to those who trained outside of Ireland and this information should be made clear to doctors from the outset.

### Limitations

The findings of this study may not be representative of the progression experiences of all non-EU doctors who have worked in Ireland for a number of reasons. An unknown number of IMGs will have arrived and left from Ireland since 2000, especially after the economic recession, which affected public sector salaries and conditions of service. Further, most of the non-EU nationals who graduated from Irish medical schools would have left after graduation or internship, and those who stayed – a sample of whom were included in this survey – may have been atypical in some way. While the low response rate is not untypical of surveys of migrant populations [28, 29], it may mean that the findings were not representative of the wider population of non-EU doctors in Ireland. As the responding sample is small, there is a possibility of bias as respondents may represent doctors who have had a poorer experience and wish to express their views. The low response rate may be due to many (an unknown number of) IMGs having possibly retained their registration with the MCI, but were not working at the time of the survey or had migrated onwards to another country.

## Conclusion

In conclusion, this study has highlighted a difference in terms of achieving career progression and training for Irish-trained non-EU doctors compared to those who trained outside of Ireland. This study has described a host country health system that relies on IMGs to fill non-training ‘service’ posts that the host country-qualified doctors will not apply for. While some of the passively recruited IMGs in this study had succeeded in obtaining specialist training, many experienced limited or stagnant career progression when compared with non-EU nationals who graduated as doctors in Ireland. This warrants further attention from a workforce planning and policy development perspective in terms of appropriately addressing the existing disparities in progression in Ireland and to ensure Ireland is meeting its obligations under the Code. At present, there is a mismatch between what migrant doctors come to Ireland for, i.e. career progression and training, with what the Irish health system can offer in terms of postgraduate training and consultant posts [26, 27, 36]. Further research to compare the experiences of the two groups in our analysis (Irish-trained non-EU doctors and those trained outside Ireland) to those of Irish and EU nationals is warranted in order to fully examine the predictors of career progression in this context.

## Abbreviations

EEA: European economic area
EU: European Union
GP: general practitioner
IMGs: international medical graduates
NCHD: non-consultant hospital doctors
OR: odds ratio

## Footnotes

1 A specialist registrar in Ireland has had several years postgraduate experience and is undergoing advanced, usually hospital based, training in a specialist field of medicine.

2 ‘Non-consultant hospital doctors’ is the term used in Ireland for junior hospital doctors. Those in the ‘Trainee Specialist Division’ are undertaking initial or higher specialist training with a view to becoming independent specialist doctors, and are registered on the ‘Specialist Division’. Doctors not in training or in independent specialist posts work in service or stand-alone posts under the supervision of hospital specialists. These are usually registered in the ‘General Division’.

3 The number of parameters that can be modelled in a logistic regression is a function of a minimum number of observations in the smallest outcome group [39]. As such, categorising career progression as a more than one level increase provided an even split between those who experienced career progression and those who did not (Table 1). Arguably, a more than one level increase also provided a more meaningful measurement of career progression as 76 % of respondents in this sample experienced at least a one level grade increase in career progression.

4 Or from year of qualification if an Irish-qualified doctor.

5 Doctors must (1) either pass or be exempt from a pre-registration examination, or (2) hold a “Certificate of Experience” or equivalent. The certificate verifies that an applicant has completed an internship which is equivalent to one completed in Ireland.
